# Supplementary material for: Pre-analytical handling conditions and protein marker recovery from urine extracellular vesicles for bladder cancer diagnosis
Source: PLoS One. 2023 Sep 7;18(9):e0291198. doi: 10.1371/journal.pone.0291198 (PMC10484439; doi:10.1371/journal.pone.0291198)
Supplement: S1 Fig — Patient urine samples were collected between day 0 and day 6 of storage. uEVs were separated using Exodisc and analyzed their sizes were assessed using nanoparticle tracking analyzer, Zetaview. (PDF) [file pone.0291198.s001.pdf]

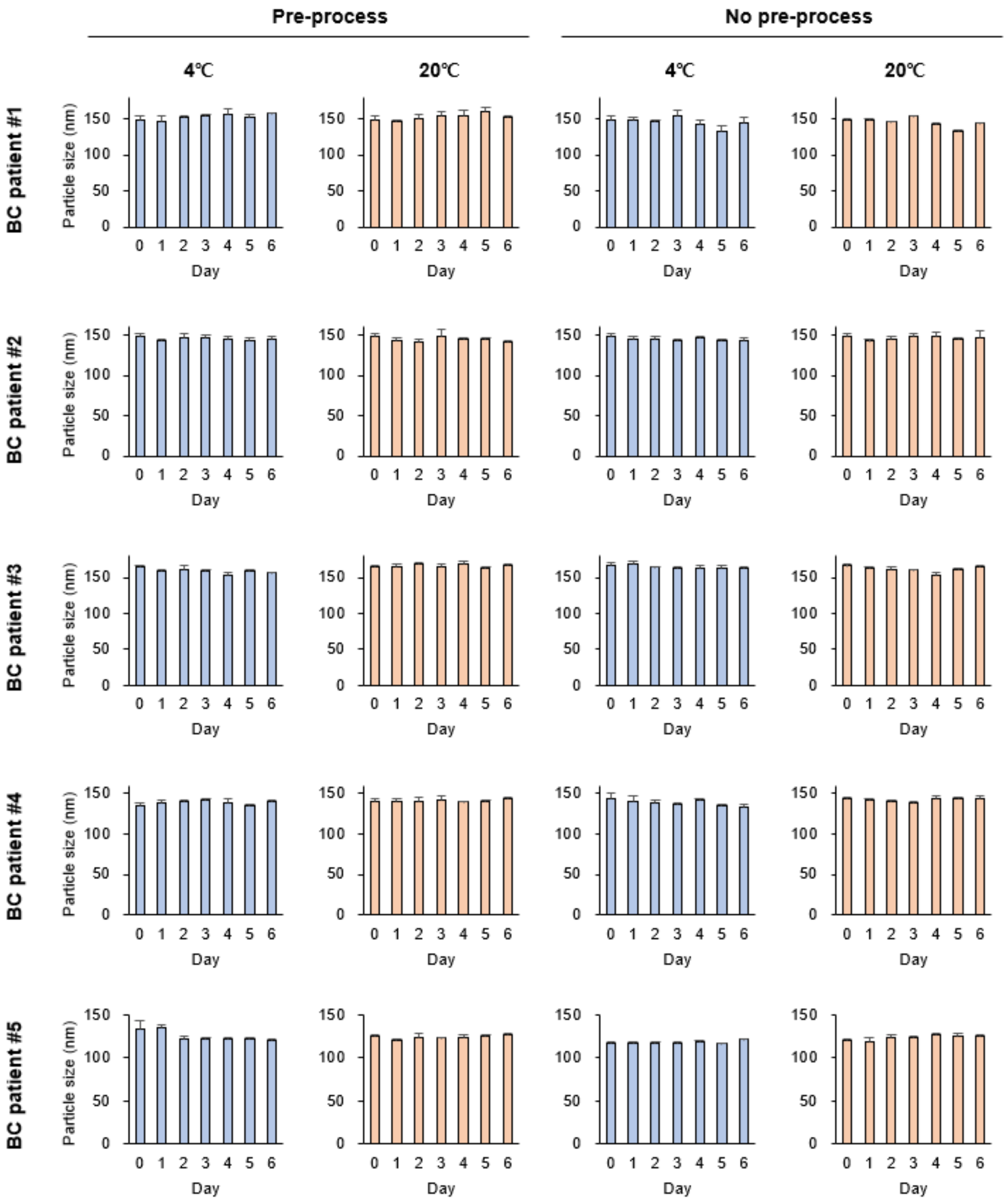

S1 Fig. Mean size of uEVs. Patient urine samples were collected between day 0 and day 6 of storage. uEVs were separated using Exodisc and analyzed their sizes were assessed using nanoparticle tracking analyzer, Zetaview.
