# Supplementary material for: Pre-analytical handling conditions and protein marker recovery from urine extracellular vesicles for bladder cancer diagnosis
Source: PLoS One. 2023 Sep 7;18(9):e0291198. doi: 10.1371/journal.pone.0291198 (PMC10484439; doi:10.1371/journal.pone.0291198)
Supplement: S2 Fig — A representative urine samples (patient#2) were collected across a span from day 0 to day 6 of storage. The subsequent step involved isolating uEVs using Exodisc, followed by subjecting them to analysis with the nanoparticle tracking analyzer, Zetaview. (PDF) [file pone.0291198.s002.pdf]

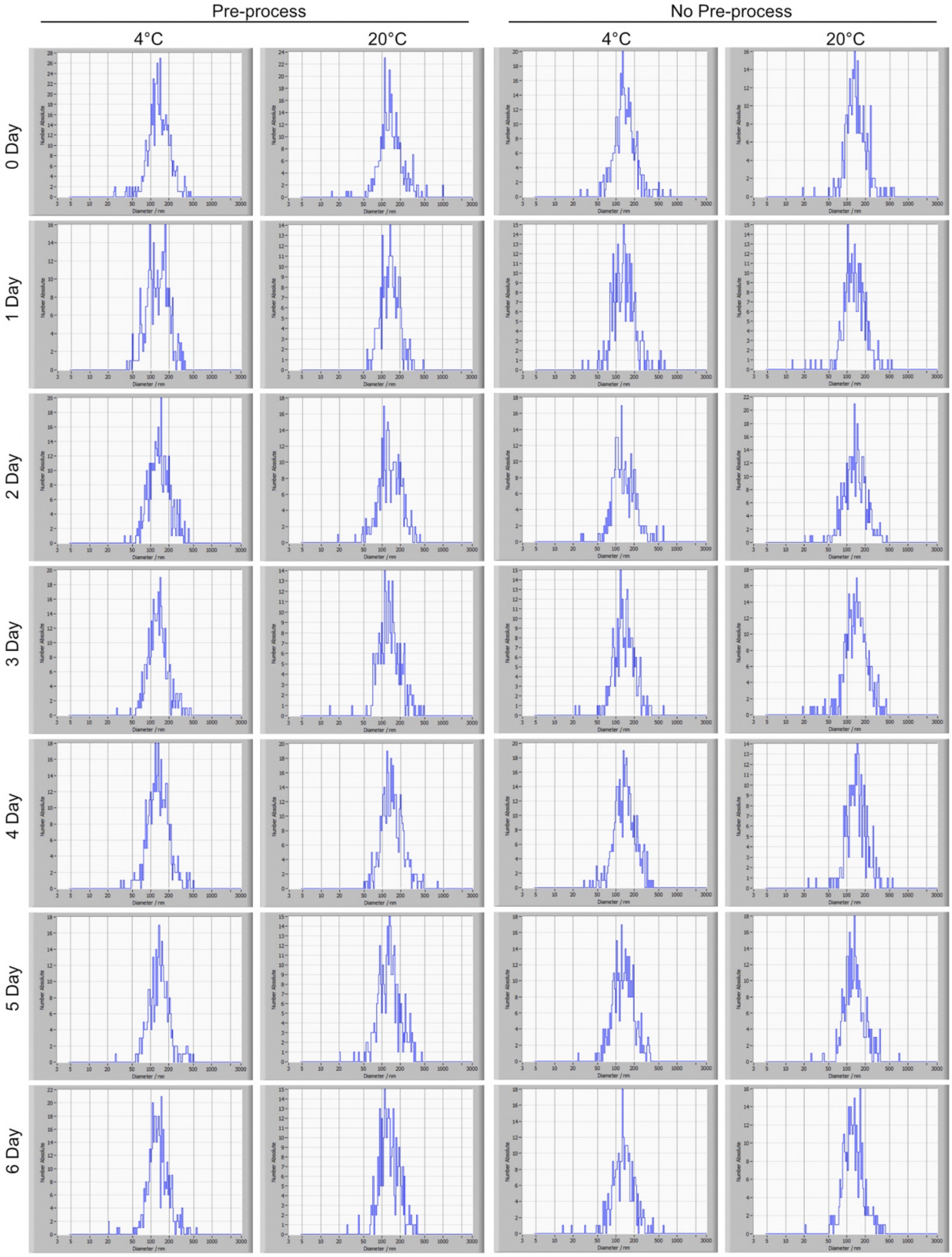

S2 Fig. Size distributions of uEVs. A representative urine samples (patient#2) were collected across a span from day 0 to day 6 of storage. The subsequent step involved isolating uEVs using Exodisc, followed by subjecting them to analysis with the nanoparticle tracking analyzer, Zetaview.
