# Supplementary material for: Pre-analytical handling conditions and protein marker recovery from urine extracellular vesicles for bladder cancer diagnosis
Source: PLoS One. 2023 Sep 7;18(9):e0291198. doi: 10.1371/journal.pone.0291198 (PMC10484439; doi:10.1371/journal.pone.0291198)
Supplement: S3 Fig — Cryo-TEM was utilized to analyze uEVs obtained from patient #1 and #3. The samples were collected on day 0 and day 6 of storage, and subsequently subjected to cryo-TEM analysis. Red arrows indicate uEVs. (PDF) [file pone.0291198.s003.pdf]

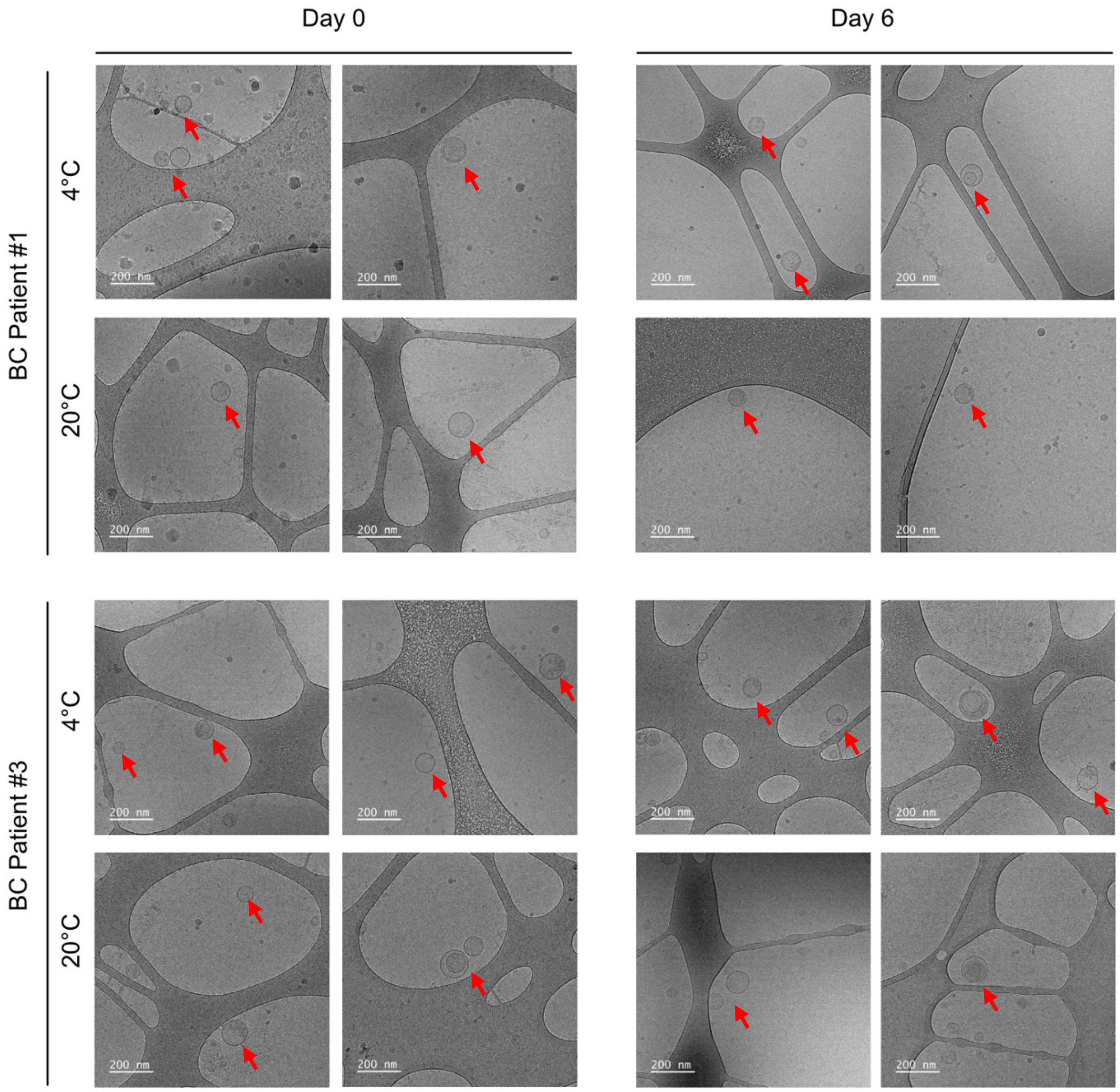

S3 Fig. Cryo-TEM analysis for EVs. Cryo-TEM was utilized to analyze uEVs obtained from patient #1 and #3. The samples were collected on day 0 and day 6 of storage, and subsequently subjected to cryo-TEM analysis. Red arrows indicate uEVs.
