# Supplementary material for: Pre-analytical handling conditions and protein marker recovery from urine extracellular vesicles for bladder cancer diagnosis
Source: PLoS One. 2023 Sep 7;18(9):e0291198. doi: 10.1371/journal.pone.0291198 (PMC10484439; doi:10.1371/journal.pone.0291198)

Pre-processing CD63 at 4°C

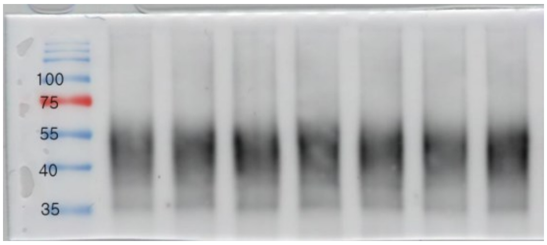

No Pre-processing CD63 at 4°C

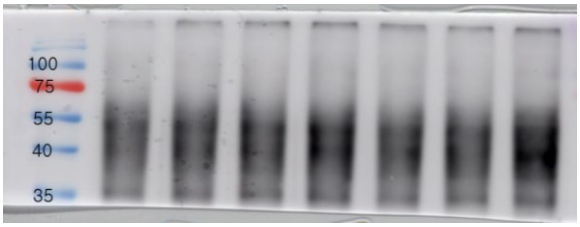

Pre-processing CD9 at 4°C

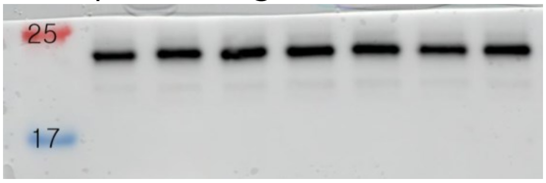

No Pre-processing CD9 at 4°C

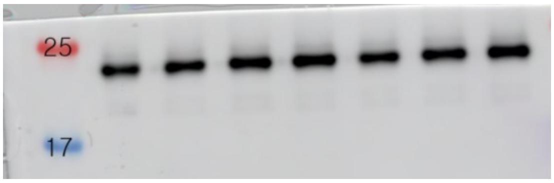

Pre-processing CANX at 4°C

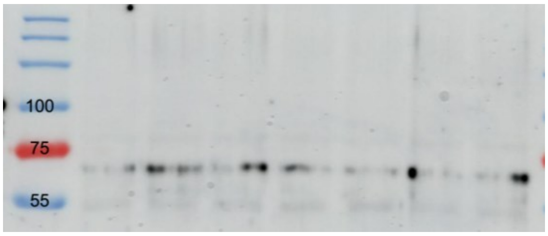

No Pre-processing CANX at 4°C

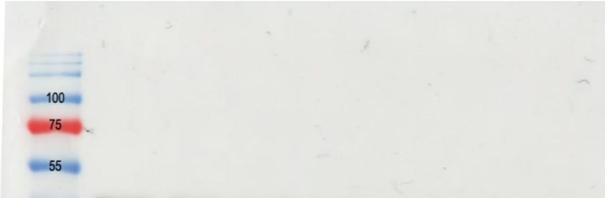

Pre-processing CD63 at 20°C

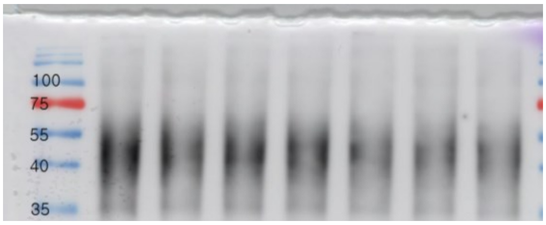

No Pre-processing CD63 at 20°C

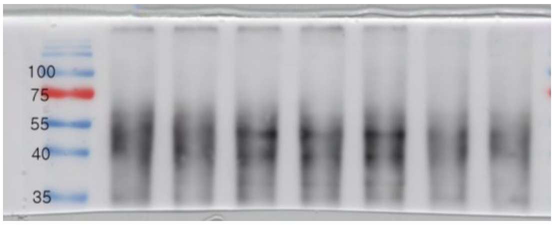

Pre-processing CD9 at 20°C

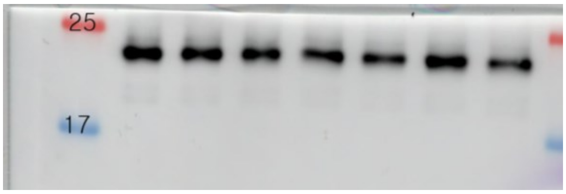

No Pre-processing CD9 at 20°C

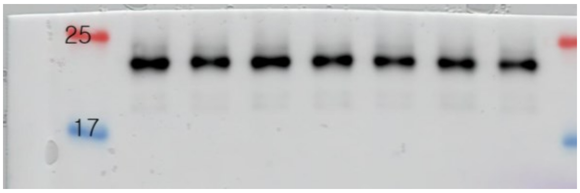

Pre-processing CANX at 20°C

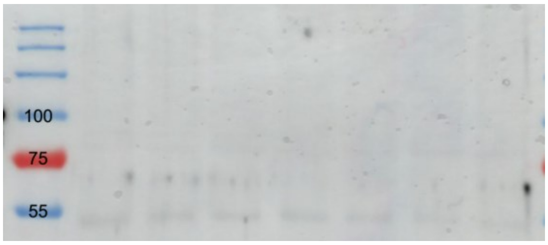

No Pre-processing CANX at 20°C

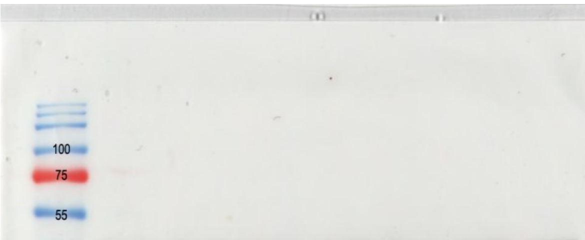

Supplement: S1 Raw images — (PDF) [file pone.0291198.s005.pdf]
