# Supplementary material for: Pre-analytical handling conditions and protein marker recovery from urine extracellular vesicles for bladder cancer diagnosis
Source: PLoS One. 2023 Sep 7;18(9):e0291198. doi: 10.1371/journal.pone.0291198 (PMC10484439; doi:10.1371/journal.pone.0291198)
Supplement: Attachment — Submitted filename: Response to Reviewers.docx [file pone.0291198.s006.docx]

Reviewer #1: In this study, the authors analyzed the quality of urinary extracellular vesicles (uEVs) stored at 20℃ or 4℃ for 0-6 days. The authors concluded that uEVs were stable when stored at 4℃ for up to 3 days. In this study, only urine samples from three bladder cancer patients were tested. It seems that the number of patients is too small to draw conclusions. For example, the amounts of A2M and CLU were different in the three patients, as shown in Figure 5 and 6. Furthermore, the effects of hematuria or pyuria are unknown. The quality of uEVs in the healthy controls is also unknown.

Authors’ response: We thank the Reviewer for the excellent suggestions. We agree that number of urine samples is not sufficient to draw a conclusion. However, it was a challenging task to prepare and analyze 42 exosome samples from three patients over a span of six days, all simultaneously, in order to minimize experimental variations. Even for an expert, conducting such experiments is not a trivial matter. Nonetheless, we were able to include two additional urine samples during the revision period, which we believe will enhance the strength of our findings. Additionally, we have added information for the information of pyruia or hematuria (Table 1). In this study, we did not use the healthy control because most uEVs from the healthy controls did not express A2M in our previous study (Lee J, Park HS, Han SR, Kang YH, Mun JY, Shin DW, Oh HW, Cho YK, Lee MS, Park J. Alpha-2-macroglobulin as a novel diagnostic biomarker for human bladder cancer in urinary extracellular vesicles. Front Oncol. 2022 Sep 13;12:976407. doi: 10.3389/fonc.2022.976407. PMID: 36176383; PMCID: PMC9513419.).

Reviewer #2: Review Comments

The manuscript titled "Pre-Analytical Handling Conditions and Protein Marker Recovery from Urine Extracellular Vesicles for Bladder Cancer Diagnosis" explores the optimal handling conditions required to maintain the stability of uEV protein markers, alpha-2-macroglobulin (A2M) and clusterin (CLU), in urine sample during transportation.

The authors discovered that storing urine at 4 °C for a maximum of three days and at 20 °C for up to 24 hours resulted in relatively stable levels of A2M and CLU, with minimal impact on analysis results. Interestingly, pre-processing did not significantly improve the preservation of protein markers in uEVs during urine storage.

This study addresses a critical issue in clinical research, as it aims to optimize the clinical protocols utilizing uEVs for diagnostic purposes. Overall, the manuscript is well-written and presents the results in an easily understandable manner.

However, there are a few points that require clarification before publication. These details are outlined below.

Authors’ response: We thank the Reviewer for the positive comments, encouragement, and support. Following the great suggestions of the Reviewer, we have revised our manuscript.

- Major points

1. In Fig 2, protein concentration seems to be decreased in pre-processing group compared to the non-processed group.
Was the analysis comparing the pre-processed group to the non-processed group conducted using an equal volume of isolated samples?

Authors’ response: Yes. We utilized urine samples of the same volume. Following pre-processing, 10 ml of urine was preserved in each tube, while 5 ml of urine was employed for uEVs isolation. As you may be aware, certain proteins have the potential to be captured by the filter during the filtration process, leading to a decline in protein concentration within the pre-processed group.

2. In Figure 3, the authors described that a Western blot was conducted using uEVs from a representative patient to examine alterations in total protein levels and specific exosome markers CD63 and CD9. The same isolated volume of uEVs was loaded for this experiment. However, Figure 2 indicates that protein concentrations decrease over time in non-processed urine stored at room temperature. Surprisingly, the Western blot results for CD9 and CD63 in all samples from non-processed urine stored at room temperature did not exhibit a significant decrease. How can this discrepancy be explained?

Authors’ response: We sincerely appreciate the insightful comments provided by the reviewer. Fig 3 in our study corresponded to the samples obtained from patient #3. As highlighted by the reviewer, we observed a decrease in the total protein content of exosomes stored at room temperature (RT). Interestingly, we found that the protein quantity did not show a strong correlation with the levels of CD63 and CD9. Nonetheless, upon comparing CD63/CD9 between day 0 and day 4 in western blot analysis, some differences were observed. However, overall, we did not observe any clear discrepancies in the western blot results, indicating no significant changes. Additionally, to investigate the possibility of morphological alterations in uEVs, we conducted cryo-transmission electron microscopy (cryo-TEM) analysis (S1 Fig). However, we did not observe any substantial morphological changes in the TEM images.

- Minor points

1. For fig 3, the size of CD63 should be specified, and it is recommended to provide complete gel images indicating the target protein size.

Authors’ response: We thank the reviewer for pointing this out. We have added whole gels for western blot in supplementary figures.

2. P6, P9, P10, P12, P13P14, P15, P16 Preprocessing were being used interchangeably, but they should be unified into one.

Authors’ response: They have unified into “pre-processing”.

3. P15, line 8 immediate → immediately
Authors’ response: We have corrected it.

Reviewer #3: In the Ms Lee and collaborators investigated the diagnostic value of alpha-2- 21 macroglobulin (A2M) and clusterin (CLU) in uEVs in respect to storage.
Concerns
TEM of uEV after different storage must be shown.
If they want to validate the protocol, more patients must be recruited

Authors’ response: We express our gratitude to the reviewer for their valuable suggestions. In response, we have incorporated the TEM image of uEV as S1 Fig in the revised manuscript. Furthermore, we have expanded our dataset by including data from two additional patients.
