# Supplementary material for: Pre-analytical handling conditions and protein marker recovery from urine extracellular vesicles for bladder cancer diagnosis
Source: PLoS One. 2023 Sep 7;18(9):e0291198. doi: 10.1371/journal.pone.0291198 (PMC10484439; doi:10.1371/journal.pone.0291198)
Supplement: Attachment — Submitted filename: Reponse to Reviewers_R2.docx [file pone.0291198.s007.docx]

**Reponse to Revewers**

Reviewer #4: The manuscript by Lee et al. looks at determining optimal conditions for handling and processing of urine EVs from bladder cancer patients. Although there is always a need to optimise practices within the liquid biopsy field the current manuscript does not adequately do this. There are a number of points which need addressing to significantly strengthen the study.

(1) Previous review has identified the need for a larger patient cohort. The authors have gone some way to address this in the current revised manuscript by increasing their cohort from 3 to 5. The major issue is that most of the conclusions are based on 2 out of 5 patients with no real clear picture. The study really needs to provide additional patients for meaningful conclusions to be made.

Authors’ response: We highly value the feedback we've received and wish to address a previous reviewer's suggestion. In the previous manuscript, we expanded our patient cohort from 3 to 5 individuals. Although we acknowledge that drawing strong conclusions from just five patients might be limited, it's crucial to understand the complexity of our experimental setup. Our approach involved analyzing 28 urinary extracellular vesicle (uEV) samples per patient across 7 time points and two temperature conditions, with and without preprocessing. This translated to a total of 140 uEV samples (5 patients × 28 samples). In the initial manuscript version, we analyzed 84 samples from 3 patients concurrently to mitigate potential biases. During the revision, we included an additional 56 uEV samples from 2 more patients.

Our core objective is to identify optimal transport conditions that maintain the integrity of uEV protein markers. The results showed that the majority of uEV protein markers remained stable even when stored at 4︒C for up to 6 days. Only one patient's samples were affected after 4 days, and another patient's after three days. Our study doesn't aim to establish statistical significance. Our focus is on defining conditions that universally preserve all samples. We intend to exclude temperatures and durations that could potentially impact the uEV protein markers. Based on our findings, we concluded that these markers remain stable for up to 3 days at 4︒C, without the necessity of pre-processing steps.

We recognize this limitation but believe our study holds significance by providing valuable insights for uEV protein marker research. The preservation of these markers is of great importance in the collection of urine samples, as it offers clinicians the convenience of shipping without the need for dry ice or extensive pre-processing. Below our revised discussion section reflects this perspective.

“This research is centered around the critical factor of sample transportation conditions, a key consideration in multicenter studies. Our findings demonstrate that the uEV-derived protein markers A2M and CLU, which hold potential for bladder cancer detection, remain stable in most patients’ uEVs when stored at 4°C for up to 6 days. Notably, only the samples from one patient demonstrated an impact after 4 days, and another patient's samples were affected after three days. The primary aim of this study isn't to establish statistical significance, but rather to identify conditions that ensure the universal preservation of all samples. Our focus was on excluding temperature and time variables that might influence uEV protein markers. This led us to conclude that these markers exhibit stability for up to three days at 4°C without necessitating pre-processing steps. While we acknowledge that drawing robust conclusions from five patients might be constrained, our method entailed an in-depth examination of 28 uEV samples per patient across 7 time points and two distinct temperature conditions, with and without pre-processing. This comprehensive approach resulted in the analysis of a total of 140 uEV samples.”

(2) The authors need to address the fact all EVs preparations, whether pre-processed or not, were ultimately “processed” as they were filtered through a 0.22 uM filter to remove aggregrates and cellular debris.

Authors’ response: We thank the reviewer for the great suggestions. We have added the description in Results section like below.

“Precisely speaking, each sample undergoes a pre-processing procedure before EV isolation. However, subsequent to urine collection, our inquiry focused on assessing whether the elimination of bacteria or debris impacts the stability of urinary EVs prior to their isolation.”

(3) Further discussion on how A2M and clusterin are likely to be presented on the surface of EVs would be helpful in understanding the influences of processing and storage on their stability.

Authors’ response: We thank the reviewer for the excellent comments. We have added a description for it in the discussion section like below.

“Although the precise underlying factor influencing the stability of A2M or CLU on EVs remains unknown, it is plausible that these proteins could be influenced by pre-processing or temperature variations, given their presence as surface proteins on EVs. the identification of these protein markers using ELISA appeared to be minimally impacted by the lysis step [18]. Hence, we hypothesize that these proteins reside on the surface of urinary EVs.”

18. Lee J, Park HS, Han SR, Kang YH, Mun JY, Shin DW, et al. Alpha-2-macroglobulin as a novel diagnostic biomarker for human bladder cancer in urinary extracellular vesicles. Front Oncol. 2022;12:976407. Epub 20220913. doi: 10.3389/fonc.2022.976407. PubMed PMID: 36176383; PubMed Central PMCID: PMCPMC9513419.

(4) Nanoparticle tracking analysis captures both number and size distribution of EVs as stated in methods. Only EV number is provided in the results. The inclusion of size distribution could be very meaningful as an additional distinguishing parameter for establishing overall changes in EV properties.

Authors’ response: We express our appreciation to the Reviewer for their valuable recommendations. Due to the relatively insignificant disparities in EV size and distribution across different conditions among patients, we did not incorporate these aspects into the initial manuscript. However, in accordance with the Reviewer's advice, we have now introduced the mean size of uEVs under each condition as Supplementary Figure 1. Additionally, we have included a representative size distribution in Supplementary Figure 2.

(5) For western blot analysis of EVs it is not clear whether equivalents numbers of EVs were loaded in each case. The authors state in the methods that EVs were quantified based on protein determination but then state that equal volumes of isolated EVs were loaded on a gel. The authors should provide total protein stains of their westerns as a means of establishing whether equivalent EVs were actually loaded. Quantifying CD9 bands may also help here as well assuming there is no variation. CD63 antibody blots are poor and difficult to interpret. Also blotting for ER-resident marker such as calnexin would establish purity of EV preparations. Blots lack negative controls.

Authors’ response: We express our gratitude to the Reviewer for their valuable insights. Given that the loaded EVs originated from an equivalent volume of EVs extracted from each urine sample, there could indeed be variations in the count of EV particles. This variability in particle numbers among the EVs is evident in Figure 4. While CD63, CD9, and CD81 are all recognized as tetraspanin EV markers, it is important to acknowledge that they can exhibit diverse expression patterns across different EV samples. In line with the Reviewer's suggestion, we have now incorporated SDS-PAGE gel and western blot analyses for calnexin. Notably, our presentation focuses exclusively on uEVs from patient samples, as uEVs from healthy individuals also express EV markers.

(6) Should highlight on EM images the actual EVs.

Authors’ response: We have indicated EVs in the EM images by arrows.

(7) The manuscript needs to be updated as text frequently refers to 3 patients rather than 5 patients.

Authors’ response: Thank the Reviewer for the kind indications. We have replaced 3 patients to 5 patients in the manuscript.

Reviewer #5: All the criticisms raised by the reviewers have been addressed by the authors. In this revised form, the quality of the paper has been significantly improved.

Authors’ response: We express our gratitude to the Reviewer for dedicating significant effort to the evaluation of our manuscript.
